# Ellagic Acid Alleviates Oxidative Stress by Mediating Nrf2 Signaling Pathways and Protects against Paraquat-Induced Intestinal Injury in Piglets

**DOI:** 10.3390/antiox11020252

**Published:** 2022-01-27

**Authors:** Yuxin Xiao, Rui Huang, Nan Wang, Yuankun Deng, Bie Tan, Yulong Yin, Ming Qi, Jing Wang

**Affiliations:** 1 College of Animal Science and Technology, Hunan Agricultural University, Changsha 410128, China; yuxinxiao@stu.hunau.edu.cn (Y.X.); huangrui@stu.hunau.edu.cn (R.H.); wangnan0317@stu.hunau.edu.cn (N.W.); dengyuankun@stu.hunau.edu.cn (Y.D.); bietan@hunau.edu.cn (B.T.); yinyulong@isa.ac.cn (Y.Y.); 2 Laboratory of Animal Nutritional Physiology and Metabolic Process, Key Laboratory of Agroecological Processes in Subtropical Region, National Engineering Laboratory for Pollution Control and Waste Utilization in Livestock and Poultry Production, Institute of Subtropical Agriculture, Chinese Academy of Sciences, Changsha 410125, China; 3 College of Resources and Evironment, University of Chinese Academy of Sciences, Beijing 100864, China; 4 Animal Nutrition and Human Health Laboratory, Hunan Provincial Key Laboratory of Animal Intestinal Function and Regulation, School of Life Sciences, Hunan Normal University, Changsha 410081, China

**Keywords:** ellagic acid, intestinal barrier, Nrf2 signaling pathway, oxidative stress, piglets

## Abstract

The gastrointestinal tract is a key source of superoxide so as to be one of the most vulnerable to oxidative stress damage. Ellagic acid (EA), a polyphenol displays widely biological activities owing to its strong antioxidant properties. Here, we investigated the protective benefits of EA on oxidative stress and intestinal barrier injury in paraquet (PQ)-challenged piglets. A total of 40 weaned piglets were randomly divided into five groups: Control, PQ, 0.005% EA-PQ, 0.01% EA-PQ, and 0.02% EA-PQ. Piglets were intraperitoneally injected with 4 mg/kg (BW) PQ or saline on d-18, and sacrificed on d-21 of experiment. EA treatments eliminated growth-check induced by PQ and increased serum superoxide dismutase (SOD) activity but decreased serum malondialdehyde (MDA) level as compared to PQ group. EA supplementation promoted Nrf2 nuclear translocation and enhanced heme oxygenase-1 (HO-1) and quinone oxidoreductase 1 (NQO1) protein abundances of small intestinal mucosa. Additionally, EA improved PQ-induced crypt deepening, goblet cells loss, and villi morphological damage. Consistently, EA increased tight junction protein expression as was evident from the decreased serum diamine oxidase (DAO) levels. EA could ameliorate the PQ-induced oxidative stress and intestinal damage through mediating Nrf2 signaling pathway. Intake of EA-rich food might prevent oxidative stress-mediated gut diseases.

## 1. Introduction

Ellagic acid (EA) is mainly found in the form of free monomers, derivatives, and complex ellagic tannins in various plants such as pomegranates, berries, and walnuts [1]. EA-rich foods have shown a broad spectrum of bioactivities in pathological conditions, including antioxidative, anti-inflammatory, and antimicrobial properties, in animal models and human studies [2]. It has been reported that EA is able to counteract the detrimental reactive oxygen (ROS) and nitrogen species (RNS), and the mechanisms rely mainly on its capacity to accept electrons and to participate in antioxidation redox reactions [3]. Since EA could either prevent oxidation by acting as free-radical scavengers or retard the oxidation process by acting through indirect pathway, it has been classified as the multi-function antioxidant. In several well-designed chronic and degenerative disease models, EA displays the capability to lower the oxidative stress markers, such as malondialdehyde (MDA) and nitric oxide, and to increase the antioxidant enzyme (e.g., catalase (CAT), superoxide dismutase (SOD)) activities [4,5,6,7,8]. Meanwhile, there is a widespread tendency to suggest that the antioxidative benefit rendered by EA in vivo is due to its gut microbial metabolites, urolithins [9,10]. In addition to the antioxidant properties, EA and urolithins also have been demonstrated to attenuate oxidative injury through regulating the apoptosis [11], autophagy [12], and mitochondrial pathways [13,14].

The GI track is a key source of ROS, yet the mucosa is a main target of various oxidants [14]. Oxidative stress is a common pathological mechanism in the pathogenesis and progression of many diseases, including gastrointestinal (GI) disorders [15]. An excess ROS would destroy the intestinal barrier structure, increase the intestinal permeability, and induce inflammation to contribute further oxidative stress. Tight junctions are intercellular adhesion complexes in epithelia, which play vital roles in establishing the intestinal barrier and the selective paracellular diffusion that is an essential process for the maintenance of gut homeostasis [16]. Urolithin A, the most powerful antioxidative microbial metabolite derived from EA, has been suggested to strongly enhance the intestinal barrier through upregulating the tight junctions proteins in Dextran Sulfate Sodium (DSS)-induced colitis mouse model [17]. They highlighted the critical requirement for aryl hydrocarbon receptor (AhR)-nuclear factor erythroid 2-related factor 2 (Nrf2) pathways in urolithin A colitis protective activity [17]. Nrf2 is an important antioxidant transcription factor, which is involved in redox-modulated signaling pathway [18,19]. Podder et al. demonstrated that EA treatment could attenuate ROS production but increase heme oxygenase-1 (HO-1) and quinone oxidoreductase 1 (NQO1) expression through activating the Nrf2 signaling pathway in paraquat (PQ)-induced human lung adenocarcinoma A549 cells [20]. However, in vivo, the beneficial effects of EA supplementation on the damaged intestinal barrier induced by oxidative stress are not clear, and its acting target still needs to be further demonstrated. Meanwhile, previous study have shown that EA can protect against oxidative stress in mice through the Nrf2/HO-1 pathway [8], but it is not common to use pigs as research subjects.

Pigs are an ideal model for the study of human gut health and pathophysiology because they share several key similarities with humans in terms of the intestinal anatomy and physiology [21,22]. In the present study, we aimed to use the piglet and a strong oxidant PQ to establish an in vivo oxidative stress model, and to exam whether EA could enhance the intestinal barrier integrity and ameliorate the systemic oxidative stress through modulating the Nrf2 pathways. The EA concentrations-gradient was set to investigate whether the beneficial effect of EA is dose dependent.

## 2. Materials and Methods

All animals used in this study were managed according to the Chinese Guidelines for Animal Welfare. The experimental protocol was approved by the Animal Care and Use Committee of the Hunan Agricultural University (Changsha, China; 2021042).

### 2.1. Animals and Experimental Design

**Figure 1:**
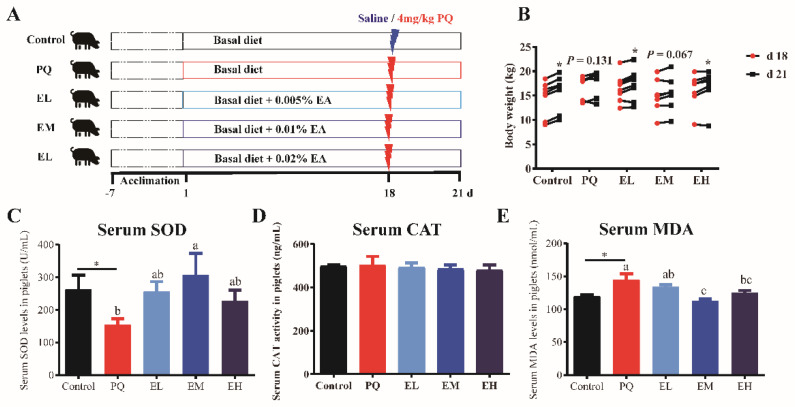
Ellagic acid supplementations alleviated the oxidative stress and growth-check of piglets challenged by paraquet. (**A**) Study design. (**B**) The BW changes of piglets on d-18 and d-21 of experiment (before and after PQ injection). (**C**–**E**) Superoxide dismutase (SOD), catalase (CAT) activities, and malondialdehyde (MDA) levels in serum of piglets. PQ = 4 mg/kg paraquet; EL = 0.005% ellagic acid + 4 mg/kg paraquet; EM = 0.01% ellagic acid + 4 mg/kg paraquet; EH = 0.02% ellagic acid + 4 mg/kg paraquet. *n* = 8. Data are shown as mean ± SEM. * in (**B**) means the difference was significant when compared to d-18. * in (**C**–**E**) means the difference was significant when compared to the control group. ns means the difference was not significant when compared to the control group. a–c Values with different lowercase letters are significantly different among PQ, EL, EM, and EH groups (*p* < 0.05).

A total of 40 piglets (Duroc × Landrace × Yorkshire) weaned at 21-d of with an average initial body weight (BW) of 8.81 ± 0.34 kg were randomly assigned to five treatments (*n* = 8) as follows: control group (control, basal diet), PQ group (PQ, basal diet), low dose EA group (EL, basal diet + 0.005% EA), middle dose EA group (EM, basal diet + 0.01% EA), and high dose EA group (EH, basal diet + 0.02% EA) (Figure 1A). The basal diets (Table 1) were formulated to meet the nutrient requirement of piglets recommended by National Research Council (2012). On day 18 of the experiment, piglets in PQ, EL, EM, and EH groups received an intra-peritoneal injection of PQ at 4 mg/kg of BW [23,24]. Piglets in the control group were injected with the same volume of isotonic saline. The three doses of EA (0.005%, 0.01%, 0.02%) were selected according to a human study that determined the EA bioavailability and bioactivity in health young volunteers [25]. The dose of EA was converted from human to pig using the equivalent calculation based on body surface area [26]. All pigs were individually housed in pens with hard plastic slatted flooring, and ad libitum access to experimental diet and water throughout the 21-d study.

The BW and feed intake were measured weekly. Average daily gain (ADG) and average daily feed intake (ADFI) were calculated as follows:

ADG = (final weight-initial weight)/time (g/d)(1)

ADFI = total feed intake/time (g/d)(2)

F/G = total feed intake/(final weight-initial weight)(3)

### 2.2. Tissue Collection and Processing

At the end of experimental period, piglets were sacrificed for serum, jejunum, and ileum tissue collection. Blood samples of 10 mL were collected aseptically from the jugular vein and then centrifuged at 3000× *g* for 10 min at 4 °C after over fasting [27]. The jejunum and ileum were dissected and rinsed thoroughly with ice-cold isotonic saline. The middle segments of the jejunum (2 cm) and ileum (2 cm) were cut and fixed in 2.5% glutaraldehyde or 4% formaldehyde for morphological and immunohistochemical analysis. Samples of the jejunal and ileal mucosa were scraped, immediately snap frozen in liquid nitrogen, and stored at −80 °C for further analysis [28].

### 2.3. Determinations of Serum SOD and CAT Activities, and MDA Levels

Superoxide dismutase (SOD), glutathione peroxidase (GSH-Px), catalase (CAT), and malondialdehyde (MDA) in serum were measured according to the manufacturing instructions contained in the porcine enzyme-linked immunosorbent assay (ELISA) kits (Jiangsu Meimian Industrial Co., Ltd., Yancheng, Jiangsu, China).

### 2.4. Determinations of Serum Diamine Oxidase and D-Lactate Concentrations

Diamine oxidase (DAO) and D-lactate (DLA) concentrations in serum were measured according to the manufacturing instructions contained in the porcine enzyme-linked immunosorbent assay (ELISA) kits (Jiangsu Meimian Industrial Co., Ltd., Yancheng, Jiangsu, China).

### 2.5. Intestinal Histological Evaluation

The segments of the jejunum and ileum fixed in 4% formaldehyde were used to determine morphology using hematoxylin-eosin staining. After dehydration, embedding, sectioning and staining, the jejunum and ileum were observed with a microscope. The villus height, crypt depth, and the number of goblet cells were measured with Case-Viewer software [29,30].

As described by German [31] and Liu et al. [32], tissue segments fixed with cold 2.5% glutaraldehyde were used for scanning and transmission electron microscopy analysis. Briefly, segments of jejunum and ileum were fixed in 2.5% glutaraldehyde for 2 h, and washed 3 × 10 min in PBS at 4 °C. Then the tissues were postfixed in 1% osmium tetroxide for 12 h at 4 °C, washed 10 min in PBS, and the PBS washing process was repeated three times. After ethanol dehydration, tert-Butyl alcohol storage, quick-drying silver paint installation, and gold-palladium coating, the tissues were examined by scanning electron microscope [29]. Moreover, after being postfixed in 1% tetroxide, the segments were dehydrated, embedded, and cut into thin sections, then stained with uranyl acetate and lead citrate for 20 min before being observed using transmission electron microscopy [33].

### 2.6. Immunofluorescence Staining

The protein abundances of occludin and claudin-3 in jejunum and ileum were detected by immuno-histochemical analysis as described by previous study [34]. Briefly, slides were blocked with 5% bovine serum albumin (BSA), and then incubated with occludin antibody (1:500; Abcam; Cambridge, UK) and claudin-3 (1:150; Abcam; Cambridge, UK) overnight at 4 °C, washed three times for 5 min in phosphate buffer saline (PBS) (pH 7.4), and then incubated with secondary antibodies, horseradish peroxidase-conjugated goat anti-rabbit IgG (1:500; Wuhan service bio technology; Wuhan, China) for 50 min in the dark. Cell nucleus were stained with 40, 6-diamidino-2-phenylindole (DAPI) for 10 min and were washed with PBS (pH 7.4) three times for 5 min per wash, then treated with a self-fluorescence quenching agent for 5 min. After sealing, images were obtained under a fluorescence microscope (NIKON ECLIPSE C1; Nikon Corporation; Tokyo, Japan).

### 2.7. Quantitative Reverse Transcription-PCR Analyses

Total RNA was extracted from jejunal and ileal mucosa according to the instructions of Trizol kit of Biyuntian, complementary deoxyribonucleic acid (cDNA) was obtained on the basis of the instruments of reverse transcription kit, and the cDNA was diluted and used as reverse transcriptase-PCR (RT-PCR) template to evaluate gene expression. The reaction system was 20 μL. RT-PCR was carried out on a Roche 48 II Fluorescent quantitative PCR apparatus, and RT-PCR conditions were as follows: 95 °C for 30 s, 95 °C for 5 s, annealing for 15 s, 72 °C for 10 s, 35 cycles, melting curve. The expression levels of target genes were quantified by comparative threshold cycle (Ct) values method, and normalized by β-actin expression levels [35,36]. Primers used in the PCR assay are listed in Table 2.

### 2.8. Western Blotting Analyses

Jejunal and ileal mucosal samples were homogenized and protein concentrations were measured using the bicinchoninic acid (BCA) Protein Concentration Assay Kit (Beyotime Institute of Biotechnology, Shanghai, China). The total proteins were extracted with RIPA lysate (Beyotime Institute of Biotechnology, Shanghai, China). Nuclear and cytosolic proteins were extracted by using a Nuclear-Cytosol Protein Extraction Kit (Beyotime Institute of Biotechnology, Shanghai, China). Proteins were separated by 6–8% sodium dodecyl sulfate-polyacrylamide gel electrophoresis (SDS-PAGE) (Beyotime Institute of Biotechnology, Shanghai, China) and transfected to PVDF membranes. Membranes were sealed for 10 min with a rapid blocking solution (Beyotime Institute of Biotechnology, Shanghai, China) at room temperature and incubated for 2 h with the primary antibodies including NQO1 (1:1000; Proteintech; Chicago, IL, USA), Nrf2 (1:600; Abcam; Cambridge, UK), HO-1(1:800; Abcam; Cambridge, UK), Recombinant Kelch Like ECH Associated Protein 1 (Keap1) (1:800; Proteintech; Chicago, IL, USA), and β-actin (1:1000; Cell Signaling Technology; Danvers, MA, USA), along with the secondary antibody horseradish peroxidase-conjugated goat anti-rabbit immunoglobulin G (1:5000; ZSGB, Biological Technology, Beijing, China). The images were detected by chemiluminescence (Millipore, Billerica, MA, USA). All protein measurements were quantified by measuring the intensity of bands using Alpha Imager 2200 Software (Alpha Innotech Corporation, San Leandro, CA, USA) and normalized to β-actin [35].

### 2.9. Statistical Analysis

Data were analyzed by using SPSS 17.0 statistical software. The differences between Ctrl and PQ group were evaluated using independent *t*-test, and the differences among PQ, EL, EM, and EH were analyzed using one-way ANOVA. Tukey-Kramer multiple comparison procedure was used for post-hoc comparisons. The Kruskal-Wallis test was used when data were not normally distributed. Besides, data in Figure 1B were performed by paired *t*-test. The *p* values less than 0.05 were considered as statistically significant. Results are presented as means ± standard error of mean (SEM).

## 3. Results

### 3.1. EA Alleviates the Oxidative Stress and Eliminates the Growth Arrest Induced by PQ in Piglets

Although there was no noticeable difference in final BW, ADG, ADFI, and F/G among five treatments (Table 3), PQ injection showed a negative effect on BW gain of piglets (Figure 1). PQ challenge induced a growth check of piglets from d-18 to d-21 of study, whereas EL and EH treatments improved the growth check (*p* < 0.05) (Figure 1B). Meanwhile, PQ induced a significant oxidative stress in piglets, which was exhibited by decreased serum SOD activities and increased serum MDA levels as compared to the control group (*p* < 0.05). Compared to the PQ group, EM remarkably enhanced serum SOD activities and decreased MDA levels (*p* < 0.05), and EH reduced serum MDA levels of piglets injected with PQ (Figure 1C,E). PQ injection and EA treatments did not affect the activities of serum CAT in piglets (Figure 1D).

### 3.2. EA Supplementation Stimulates the Nrf2-HO1/NQO1 Signaling Pathway in Small Intestine of Piglets

**Figure 2:**
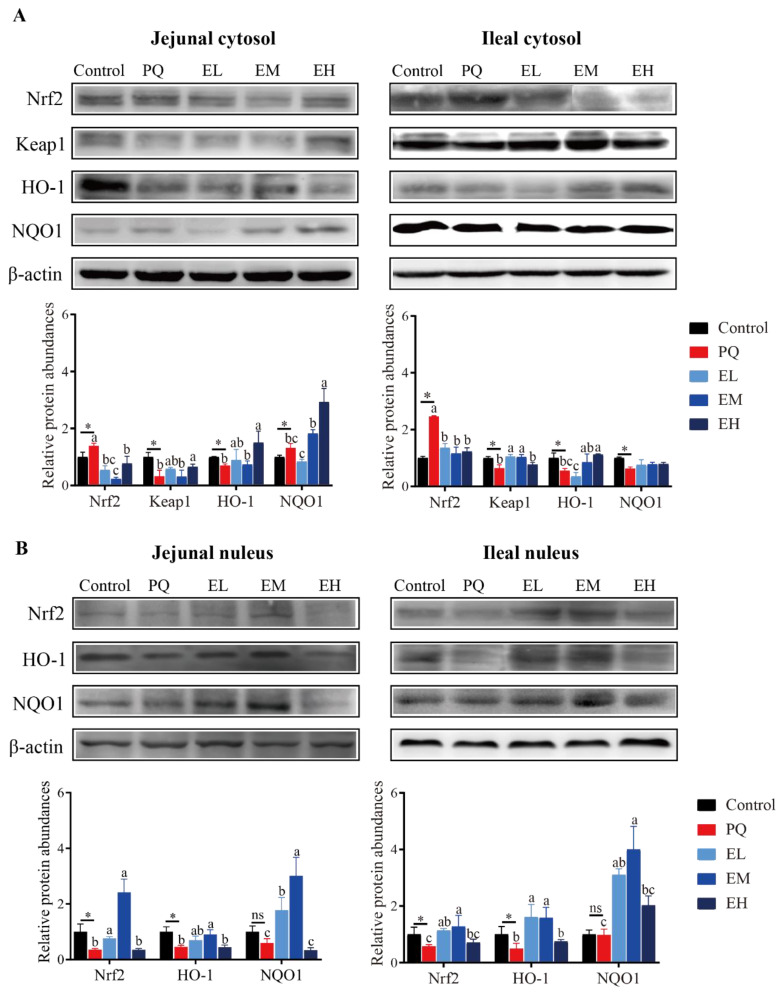
Protein abundances of Nrf2 signaling pathway of cytosol and nucleus of jejunal and ileal mucosa in piglets. (**A**) Nuclear factor erythroid 2-related factor 2 (Nrf2), Recombinant Kelch Like ECH Associated Protein 1 (Keap1), heme oxygenase-1 (HO-1), and quinone oxidoreductase 1 (NQO1) protein abundances in cytosol of jejunal and ileal mucosa. (**B**) Nrf2, HO-1, and NQO1 protein abundances in nucleus of jejunal and ileal mucosa. PQ = 4 mg/kg paraquet; EL = 0.005% ellagic acid + 4 mg/kg paraquet; EM = 0.01% ellagic acid + 4 mg/kg paraquet; EH = 0.02% ellagic acid + 4 mg/kg paraquet. *n* = 8. Data are shown as mean ± SEM. * means the difference was significant when compared to the control group. ns means the difference was not significant when compared to the control group. ^a–c^ Values with different lowercase letters are significantly different among PQ, EL, EM, and EH groups (*p* < 0.05).

Nrf2 has been reported to involve in the redox-modulated cell signaling. In order to investigate whether EA eliminated the oxidative stress via regulating Nrf2 signaling pathway, we detected the mRNA and protein expressions of NQO1, HO-1, Nrf2, and Keap1 in small intestinal mucosa (Figure 2). Compared to the control group, PQ decreased the ileal mucosal Nrf2, Keap1, and HO-1 mRNA relative expressions but increased the jejunal mucosal HO-1 and NQO1 mRNA relative expressions (*p* < 0.05). EA treatments had no effect on the mRNA expression levels of Nrf2 signaling pathway in jejunal mucosa (Figure S1A). However, compared to the PQ group, EM increased HO-1 mRNA levels, and EH up-regulated Nrf2, Keap1, and HO-1 mRNA levels in ileal mucosa (*p* < 0.05) (Figure S1B).

Nrf2 is blocked in the cytosol by its inhibitor Keap1 when cells stay at equilibrium redox state. It is released from Keap1 and translocated into the nucleus, where it binds with elements participated in transcription of genes coding antioxidant enzymes, when the redox state is out of balance [37]. In jejunal mucosa, EA stimulated the Nrf2-Keap1-HO1/NQO1 signaling pathway by enhancing Nrf2 protein levels in the EL and EM groups, and enhancing HO1 protein levels in the EH group. In ileal mucosa, EA stimulated Nrf2 signaling pathway mainly by inducing Keap1 protein expressions (Figure S1C,D). We also detected the relative protein levels of Nrf2, Keap1, NQO1, and HO-1 in cytosol or nucleus of jejunal and ileal mucosa (Figure 2A,B). Compared with the control group, PQ increased Nrf2 but decreased Keap1 protein levels in cytoplasm of jejunal and ileal mucosa, as well as decreased Nrf2 protein levels in nucleus of jejunal and ileal mucosa. EL and EM treatments promoted the nuclear transportation of Nrf2 protein exhibited by increasing its levels in nucleus of jejunal and ileal mucosa. Meanwhile, PQ decreased HO-1 protein abundances in jejunal and ileal cytoplasm and nucleus, as well as decreased NQO1 protein abundances in ileal cytoplasm while increased that in jejunal cytoplasm. In jejunal and ileal mucosa, EL and EM treatments increased NQO1 protein expressions in nucleus, and EH increased HO-1 protein expressions in cytoplasm.

### 3.3. EA Supplementation Improves the Intestinal Morphology of PQ-Challenged Piglets

**Figure 3:**
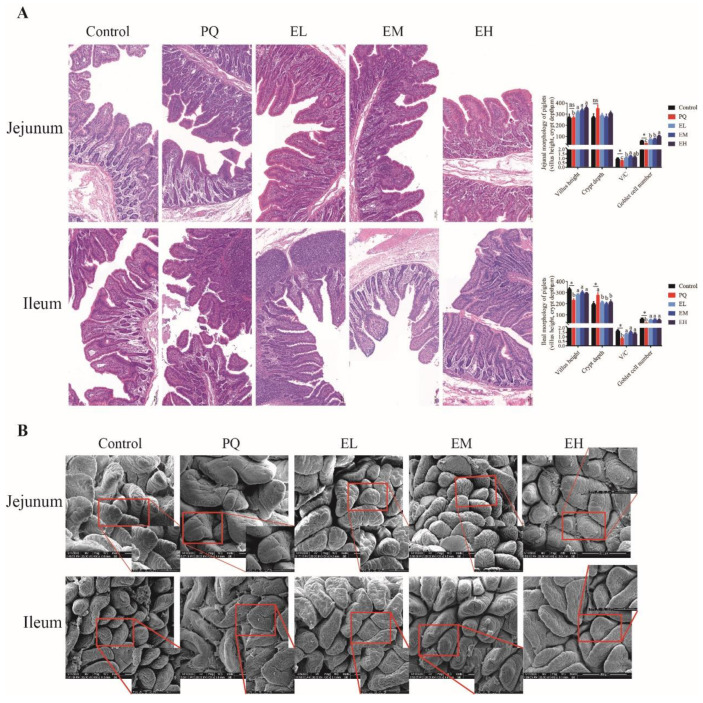
Intestinal mucosal morphology of piglets. (**A**) The histological representative images and histogram of morphological parameters of jejunal and ileal mucosa (magnification 200×). (**B**) Scanning electron microscopy analysis of jejunal and ileal mucosa (magnification 400×, 1000×). PQ = 4 mg/kg paraquet; EL = 0.005% ellagic acid + 4 mg/kg paraquet; EM = 0.01% ellagic acid + 4 mg/kg paraquet; EH = 0.02% ellagic acid + 4 mg/kg paraquet. *n* = 8. Data are shown as mean ± SEM. * means the difference was significant when compared to the control group. ns means the difference was not significant when compared to the control group. ^a–c^ Values with different lowercase letters are significantly different among PQ, EL, EM, and EH groups (*p* < 0.05).

Oxidative stress induces gut epithelial barrier disruptions, which is associated with the intestinal morphological damage and the increased gut permeability [38]. As shown in Figure 3, PQ decreased the ratio of villus height to crypt depth (V/C) and the number of goblet cells in jejunum and ileum, while the above two indications increased in EA (*p* < 0.05). (Figure 3A). Compared with the control group, PQ caused a decrease of villi density and villi folds in the jejunum and ileum, while supplementation of EA induced an increase in villi density and villi folds (Figure 3B). These results indicated that EA might recover the intestinal morphological injury of PQ-challenged piglets.

### 3.4. EA Supplementation Maintains the Structure of Tight Junction and Decreases the Permeability of Intestinal Barrier in PQ Challenged Piglets

**Figure 4:**
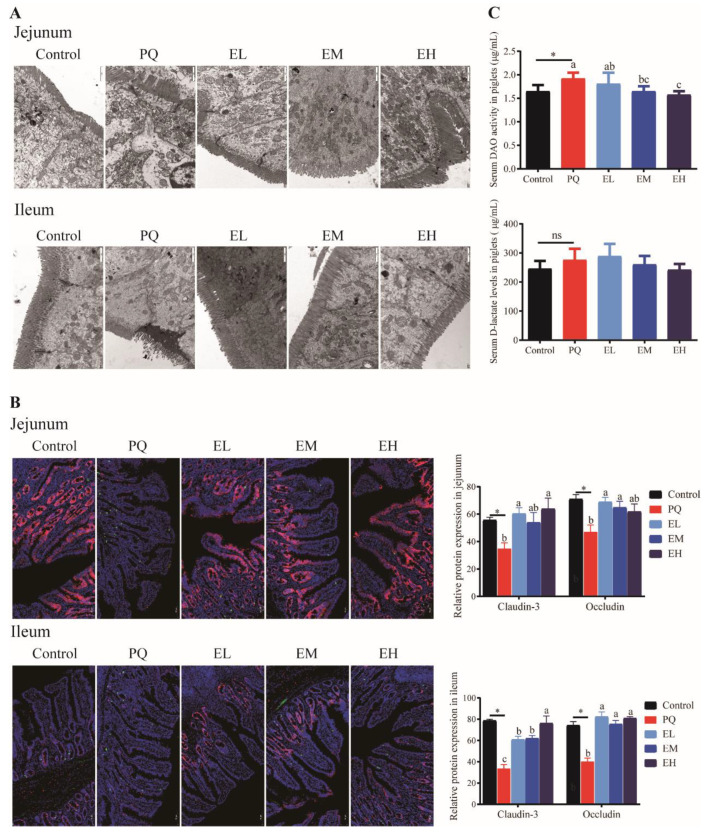
Tight junction structure and permeability of jejunal and ileal mucosa of piglets. (**A**) Representative electron photomicrograph and quantification of microvillus and tight junction; the microvillus and the tight junction are indicated by the black arrow and the white arrow, respectively. (**B**) The expression of tight junction proteins (Red: Claudin3; green: Occludin) in jejunal and ileal mucosa. (**C**) Serum diamine oxidase (DAO) and D-lactate (DLA) levels. PQ = 4 mg/kg paraquet; EL = 0.005% ellagic acid + 4 mg/kg paraquet; EM = 0.01% ellagic acid + 4 mg/kg paraquet; EH = 0.02% ellagic acid + 4 mg/kg paraquet. *n* = 8. Data are shown as mean ± SEM. * means the difference was significant when compared to the control group. ns means the difference was not significant when compared to the control group. ^a–c^ Values with different lowercase letters are significantly different among PQ, EL, EM, and EH groups (*p* < 0.05).

Because the tight junction plays a key role in maintaining the intestinal mucosal barrier integrity and the impermeability [39], we also evaluated the structure and protein abundances of tight junction (Figure 4A,B). Piglets in PQ group were observed to have shorter microvilli and a larger gap of enterocytes in jejunum and ileum as compared to the control group. Conversely, EA treatments displayed longer microvilli and restored intact tight junction protein structures in the jejunum and ileum (Figure 4A). Consistently, PQ significantly decreased the protein abundances of claudin-3 and occludin in jejunum and ileum as compared to control group (*p* < 0.05), whereas EA treatments enhanced these protein expressions of in jejunum and ileum (*p* < 0.05) (Figure 4B). Compared to the control group, PQ challenge increased DAO levels in serum (*p* < 0.05). Compared to the PQ group, EM and EH groups significantly reduced serum DAO levels (*p* < 0.05) (Figure 4C). These results implied that EA supplementations could maintain the structure of tight junction and decrease the permeability of intestinal barrier in PQ challenged piglets.

## 4. Discussion

Pigs are subjected to various adverse stimuli during the entire production cycle. These adverse stimuli eventually translate into imbalanced redox levels, which has limited the development of high-efficiency and high-quality swine industry. Numerous studies have reported the anti-oxidative benefits of EA or foods enrich with EA from pomegranates, barriers, and walnuts on various metabolic diseases (rodent model and in vitro model) [11,40,41], but few studies considered the direct effect of EA on oxidative damage of intestinal barrier in vivo. Our current study found that EA could alleviate the oxidative damage of intestinal barrier in piglets challenged by PQ, and these effects were associated with the key regulatory factor Nrf2 signaling pathway.

Piglets post weaning having immature intestine and immune systems are susceptible to oxidative stress attack, which has a direct negative impact on piglets growth performance [42]. Previous studies have shown that oxidative stress (e.g., piglets feeding with oxidized fish oil [43], peroxidized-lipids [44] or D-galactose containing diets [45], or received an injection of diquat [43,46]) decreases the feed intake and the final BW, and slows down the weight gain of piglets. Despite the fact that in our study PQ challenge did not affect the final BW, ADG, and ADFI during the whole experimental period, the PQ caused the growth-check after injection as compared to the control group. EA treatment, especially low dose and high dose EA supplementation, eliminated this growth-check induced by PQ, which might be as a result of EA antioxidative properties. Many studies have shown that EA, as a strong oxidant that can effectively scavenge free radicals, plays an important regulatory role in maintaining redox homeostasis and oxidative stress injury repair [47]. MDA, as biomarkers of cellular oxidative stress, is an end product of lipid peroxidation [48], and SOD and CAT provide the major antioxidant defense against reactive species [15]. EA supplementations in this study decreased the serum MDA levels but increased SOD activities of PQ-induced piglets, which is consistent with the previous studies [49,50,51,52]. The expression of defense genes coding antioxidant proteins is mediated by Nrf2-Keap1 pathway, which is switched on or off directly by ROS so as to be a signaling pathway committed to the oxidant elimination [53]. An increasing number of studies demonstrate that in oxidative stress induced diseases (such as diabetes induced tissue damage, memory impairment, and hepatic injury, but not gut diseases-related evidences), EA elevates the activities SOD and glutathione (GSH) and declines the MDA levels by increasing the nuclear translocation of Nrf2, thereby protecting cells from the free radical damage [54,55,56,57,58]. In the present study, EA treatments did not change the gene expression of *nqo1* in the small intestine, but up-regulated *ho-1*, *nrf2* and *keap1* mRNA levels of ileal mucosa of piglets. Consistently, EA promoted Nrf2 nuclear translocation and enhanced the HO-1 and NQO1 protein abundances in both cytoplasm and nucleus of jejunal and ileal mucosa. Moreover, many researchers suggest that the bioactivities of EA at least partially depend on its intestinal microbial metabolites, urolithins. Besides, a previous study suggested that the antioxidant effect of EA on gastrointestinal tract is closely related to its metabolites, urolithins [59]. The microbial community loads and composition changes along the gastrointestinal tract, and this might cause the results in jejunum and ileum having slightly differences. Altogether, our results indicated EA could alleviate the oxidative stress of PQ-induced piglets, and this function might be associated with the activation of Nrf2-Keap1 signaling pathway.

In addition, Nrf2 plays a regulatory role in intestinal barrier integrity [60,61]. In the present study, we also observed that EA administrations alleviated microvilli shedding and crypt hyperplasia, as well as increased the number of goblet cells in piglets injected with PQ, which is consistent with previous studies [51]. Oxidative stress can cause the intestinal barrier injury mainly manifested as villi shorting, crypt hyperplasia, and goblet cells apoptosis, which contribute further to a reduced ability for nutritional absorption and presentation of pathogens [62,63]. Maintaining intestinal integrity is a prerequisite to ensure the intestinal barrier function [64]. Tight junction proteins, an important part of the intestinal barrier, have been shown to be enhanced by urolithin A via the Nrf2-Keap1 signaling pathway [17,65]. Here, our results showed that EA supplementations alleviated the occludin and claudin-3 proteins deficiency in small intestinal mucosa caused by PQ challenge. Occludin and claudin-3, as the tight junction components between intestinal epithelial cells, were responsible for the barrier tightness [66,67,68]. Similarly, transmission electron microscope results suggested that the intestinal tight junction structures in the EA group were more complete and clearer than these in PQ group. Serum DAO and DLA levels are two bio-markers of the intestinal barrier permeability [69]. EA treatments significantly reduced the serum level of DAO and tended to decrease the serum level of DLA, which is consistent with the decreased intestinal permeability. Data from this study supported that EA could restore the damaged intestinal morphology and tight junction structure, thereby reducing intestinal barrier permeability.

## 5. Conclusions

In summary, our results confirm that the protective effect of EA against PQ-induced oxidative damage in piglets and prove the protective mechanism may rely on two approaches: (1) Maintaining REDOX balance mediated by nuclear transport of Nrf2. (2) Improving intestinal mucosal barrier integrity by up-regulating tight junction protein expressions. Further researches are needed on the specific mechanism of Nrf2 regulating intestinal barrier. Notably, moderate intake of EA (0.001% EA) should have the best beneficial effect in weaned piglets injected with PQ. EA, as a powerful plant-based antioxidant, has the advantages of high safety and low cost, and our study indicated its application prospect in swine production, as well as provided an appropriate additive dose of EA in piglets. However, the application of EA in animal production are needed to be verified by more animal models and clinical trials.

## Figures and Tables

**Table 1:** Ingredients and composition of the basal diet (%).

Ingredients	
Corn	32.50
Extruded corn	30.00
Soybean meal	10.14
Fermented soybean meal	8.00
Fish meal	5.00
Whey powder	6.00
Mineral meal	0.20
Calcium bicarbonate	0.40
Soybean oil	2.00
Glucose	3.00
L-lysine (98%)	0.55
D,L-methionine	0.07
L-threonine	0.20
L-tryptophan (98%)	0.04
Organic acid Calcium	0.60
Choline chloride	0.01
Antioxidant	0.05
Mineral premix ^1^	0.15
Vitamin premix ^2^	0.04
ZnO	0.40
Acidifier	0.70
Total	100.00
Nutrient composition ^3^	
Digestive energy, MJ/kg	15.80
Crude protein	16.20
Calcium	0.72
Total phosphorus	0.66
Total Lysine	1.46

^1^ Mineral premix provided for 1 kg of completed diet: Zn (ZnO), 50 mg; Cu (CuSO_4_), 20 mg; Mn (MnO), 55 mg; Fe (FeSO_4_), 100 mg; I (KI), 1 mg; Co (CoSO_4_), 2 mg; Se (Na_2_SeO_3_), 0.3 mg. ^2^ Vitamin premix provided for 1 kg completed diet: vitamin A, 8255 IU; vitamin D3, 2000 IU; vitamin E, 40 IU; vitamin B1, 2 mg; vitamin B2, 4 mg; pantothenic acid, 15 mg; vitamin B6, 10 mg; vitamin B12, 0.05 mg; nicotinic acid, 30 mg; folic acid, 2 mg; vitamin K3, 1.5 mg; biotin, 0.2 mg; choline chloride, 800 mg; and vitamin C, 100 mg. ^3^ Calculated values.

**Table 2:** Primer sequences ^1^.

	Forward Primer 5′ to 3′	Reverse Primer 5′ to 3′	Accession Number
NQO1	CCAGCAGCCCGGCCAATCTG	AGGTCCGACACGGCGACCTC	NM_001159613.1
Nrf2	CACCACCTCAGGGTAATA	GCGGCTTGAATGTTTGTC	XM_005671981.3
Keap1	AGCTGGGATGCCTCAGTGTT	AGGCAAGTTCTCCCAGACATTC	NM_001114671.1
HO-1	AGCTGTTTCTGAGCCTCCAA	CAAGACGGAAACACGAGACA	NM_001004027.1
β-actin	CTGCGGCATCCACGAAACT	AGGGCCGTGATCTCCTTCTG	XM_003124280.5

^1^ NQO1 = Quinone oxidoreductase 1; Nrf2 = Nuclear factor erythroid 2-related factor 2; Keap1 = Recombinant Kelch Like ECH Associated Protein 1; HO-1 = Heme oxygenase-1.

**Table 3:** Growth performance of piglets ^1,2^.

	Control	PQ	EL	EM	EH	*P*_t_	*P_EA_*
Initial BW (kg)	8.81 ± 0.34	8.82 ± 0.30	8.81 ± 0.32	8.81 ± 0.33	8.81 ± 0.33	1.000	1.000
Final BW (kg)	15.60 ± 1.41	17.42 ± 1.16	17.32 ± 1.11	15.41 ± 1.34	16.71 ± 1.40	0.352	0.661
ADG (g/d)	375.00 ± 60.00	413.09 ± 47.89	405.06 ± 45.39	372.22 ± 39.91	385.37 ± 57.23	0.630	0.938
ADFI (g/d)	481.90 ± 94.21	601.51 ± 43.94	689.70 ± 47.09	523.10 ± 86.95	699.25 ± 69.57	0.301	0.200
F/G	2.72 ± 0.24	2.83 ± 0.37	2.58 ± 0.41	2.79 ± 0.18	2.33 ± 1.77	0.816	0.736

^1^ PQ = 4 mg/kg paraquet; EL = 0.005% ellagic acid + 4 mg/kg paraquet; EM = 0.01% ellagic acid + 4 mg/kg paraquet; EH = 0.02% ellagic acid + 4 mg/kg paraquet. BW = Body weight; ADFI = Average daily feed intake; ADG = Average daily gain; F/G = ADFI/ADG. ^2^ *n* = 8; data are presented as means ± SEM; *P*_t_: Control vs. PQ; *P_EA_*: PQ vs. (EL, EM, EH).

## Data Availability

Data are contained within the article.

## Supplementary Materials

The following supporting information can be downloaded at: https://www.mdpi.com/article/10.3390/antiox11020252/s1, Figure S1: Relative gene and protein expressions of Nrf2 signaling pathway in jejunal and ileal mucosa of piglets.

## Abbreviations

EA	Ellagic acid
PQ	paraquet
EL	low dose EA group;
EM	middle dose EA group
EH	high dose EA group
SOD	superoxide dismutase
MDA	malondialdehyde
HO-1	heme oxygenase-1
NQO1	quinone oxidoreductase 1
ROS	reactive oxygen
RNS	nitrogen species
CAT	catalase
GI	gastrointestinal
DSS	Dextran Sulfate Sodium
AhR	hydrocarbon receptor; BW, body weight
ADG	Average daily gain
ADFI	average daily feed intake
GSH-Px	glutathione peroxidase;
ELISA	enzyme-linked immunosorbent assay
DAO	Diamine oxidase
DLA	D-lactate
BSA	Bovine Serum Albumin
DAPI	40, 6-diamidino-2-phenylindole
cDNA	complementary deoxyribonucleic acid
Ct	threshold cycle
BCA	Bicinchoninic Acid
SDS-PAGE	sodium dodecyl sulfate-polyacrylamide gel electrophoresis
PVDF	poly vinylidene fluoride
V/C	villus height to crypt depth
GSH	glutathione
