# Supplementary material for: Ellagic Acid Alleviates Oxidative Stress by Mediating Nrf2 Signaling Pathways and Protects against Paraquat-Induced Intestinal Injury in Piglets
Source: Antioxidants (Basel). 2022 Jan 27;11(2):252. doi: 10.3390/antiox11020252 (PMC8868335; doi:10.3390/antiox11020252)
Supplement: Supplementary file 1 [file antioxidants-11-00252-s001.zip › antioxidants-1513366-supplementary.pdf]

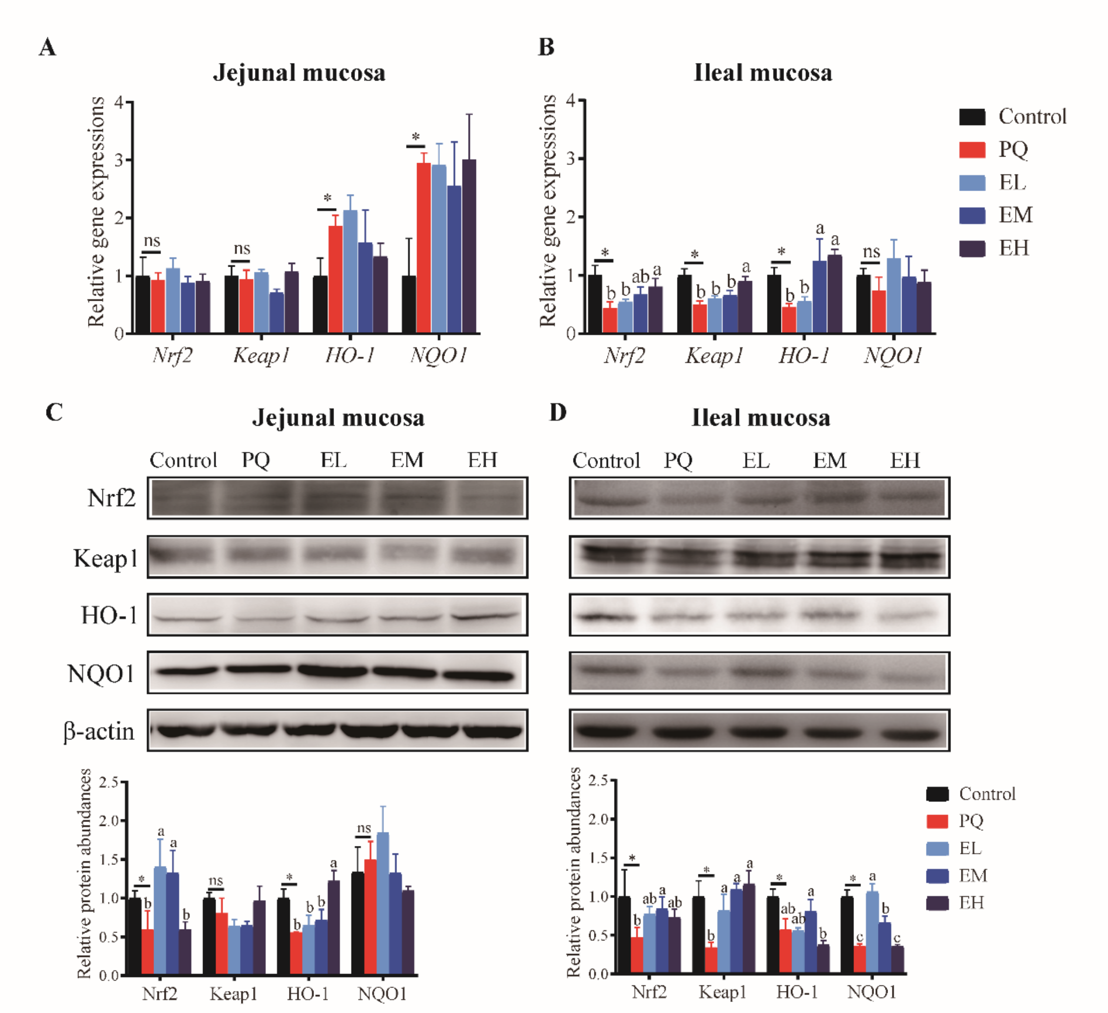

Figure S1. Relative gene and protein expressions of Nrf2 signaling pathway in jejunal and ileal mucosa of piglets. Nuclear factor erythroid 2-related factor 2 (Nrf2), Recombinant Kelch Like ECH Associated Protein 1 (Keap1), heme oxygenase-1 (HO-1) and quinone oxidoreductase 1 (NQO1) gene expressions of jejunal (A) and ileal mucosa (B). Nrf2, Keap1, HO1 and NQO1 protein expressions of jejunal (C) and ileal mucosa (D). PQ = 4 mg/kg paraquet; EL = 0.005% ellagic acid + 4 mg/kg paraquet; EM = 0.01% ellagic acid + 4 mg/kg paraquet; EH = 0.02% ellagic acid + 4 mg/kg paraquet. n = 8. Data are shown as mean ± SEM. * means the difference was significant when compared to the control group. n.s. means the difference was not significant when compared to the control group. [a-c] Values with different lowercase letters are significantly different among PQ, EL, EM, and EH groups ($P < 0.05$).
